# Molecular and clinicopathological characteristics of *ROS1*‐rearranged non‐small‐cell lung cancers identified by next‐generation sequencing

**DOI:** 10.1002/1878-0261.12789

**Published:** 2020-09-14

**Authors:** Meiying Cui, Yuchen Han, Pan Li, Jianying Zhang, Qiuxiang Ou, Xiaoling Tong, Ruiying Zhao, Nan Dong, Xue Wu, Wencai Li, Guozhong Jiang

**Affiliations:** ^1^ Department of Pathology The First Affiliated Hospital of Zhengzhou University Zhengzhou University China; ^2^ Department of Pathology Shanghai Chest Hospital China; ^3^ Institute of Medical and Pharmaceutical Sciences Zhengzhou University China; ^4^ Translational Medicine Research Institute Geneseeq Technology Inc. Toronto Canada

**Keywords:** crizotinib, gene fusion, next‐generation sequencing, non‐small‐cell lung cancer, *ROS1*

## Abstract

*ROS1* gene rearrangements have been reported in diverse cancer types including non‐small‐cell lung cancer (NSCLC), and with a notably higher prevalence in lung adenocarcinoma. The tyrosine kinase inhibitors, crizotinib, lorlatinib, and entrectinib, have demonstrated favorable efficacy in treating *ROS1‐*rearranged NSCLCs. Herein, we retrospectively reviewed 17 158 NSCLC patients whose tumor specimen and/or circulating cell‐free DNA underwent comprehensive genomic profiling. A total of 258 unique patients were identified with *ROS1* rearrangements, representing an overall prevalence of approximately 1.5% of *ROS1* fusions in newly diagnosed and relapsed NSCLC patients. *CD74* (38%) was the most common fusion partner of *ROS1*, followed by *EZR* (13%), *SDC4* (13%), *SLC34A2* (10%), and other recurrent fusion partners with lower frequencies, including *TPM3*, *MYH9*, and *CCDC6*. Variant breakpoints occurred in *ROS1* introns 33 (37%), 31 (25%), 32 (17%), and 34 (11%) with no obvious hotspots. *CD74* (63%) and *EZR* (50%) were more frequently fused to *ROS1* intron 33 than other introns, while *ROS1* intron 31 was most frequently fused with *SDC4* (79%) and *SLC34A2* (81%). Crizotinib progression‐free survival (PFS) was not significantly different between fusion variants involving breakpoints in different *ROS1* introns, nor was there a significant difference in PFS between *CD74‐ROS1* and non‐*CD74*‐*ROS1* groups of patients. Furthermore, *TP53* was most frequently mutated in patients who progressed on crizotinib, and *TP53* mutations were significantly associated with shorter crizotinib PFS. *ROS1* mutations, including G2032R, were observed in approximately 33% of post‐crizotinib samples. Collectively, we report the prevalence of *ROS1* fusions in a large‐scale NSCLC population and the efficacy of crizotinib in treating patients with *ROS1‐*rearranged NSCLC.

## Graphical abstract

Using next‐generation sequencing, this study found that the prevalence of *ROS1* fusions in a large cohort of non‐small‐cell lung cancers (NSCLCs) was 1.5%, including most frequent fusion partners and rare *ROS1* fusion pairs. Crizotinib demonstrated robust response in treating patients with *ROS1*‐rearranged NSCLC. *TP53* mutations in *ROS1*‐positive patients were significantly associated with shorter crizotinib progression‐free survival.

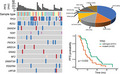

**Abbreviations**
AF: allele frequency
ALK: anaplastic lymphoma kinase
CNS: central nervous system
FFPE: formalin‐fixed paraffin‐embedded
LUAC: lung adenocarcinoma
NGS: next‐generation sequencing
NSCLC: non‐small‐cell lung cancer
ORR: objective response rate
PFS: progression‐free survival
TKI: tyrosine kinase inhibitor

## 1 Introduction

The proto‐oncogene, *ROS1*, which is mutated in multiple solid tumors and results in the dysfunction of a tyrosine kinase‐mediated signaling pathway, was identified specifically in non‐small‐cell lung cancer (NSCLC) patients in 2007 in cases where *ROS1* rearrangements occurred between the fusion partner, solute carrier family 34 member 2 gene (*SLC34A2*) and CD74 molecule gene (*CD74*) [1]. To date, more than 20 *ROS1* fusion partners have been identified, including the syndecan 4 gene (*SDC4*), the tropomyosin 3 gene (*TPM3*), the ezrin gene (*EZR*), and the leucine‐rich repeats and immunoglobulin‐like domain 3 gene (*LRIG3*) [2, 3]. Notably, the number of *ROS1* fusion partners identified continues to increase due to the adoption of next‐generation sequencing (NGS) for genetic testing [4].

Crizotinib, an anaplastic lymphoma kinase (ALK)/ROS1/MET proto‐oncogene, receptor tyrosine kinase (MET) inhibitor, was the first targeted agent approved by the US Food and Drug Administration for the treatment of advanced *ROS1*‐rearanged NSCLC. Such approval was based on evidence from the expansion cohort of the phase I crizotinib study (PROFILE 1001) that demonstrated an objective response rate (ORR) of 72% and a median progression‐free survival (mPFS) of 19.2 months in advanced *ROS1*‐positive NSCLCs [5], despite the fact that most patients eventually experienced disease relapse due to acquired resistance. Other tyrosine kinase inhibitors (TKIs), including ceritinib [6], lorlatinib [7], and entrectinib [8], have recently shown favorable clinical responses in the *ROS1*‐rearranged NSCLC patient population, including in patients with brain metastases at baseline [9], thus demonstrating superior blood–brain barrier penetration compared to crizotinib. Herein, we aimed to identify the landscape of *ROS1* gene fusions in Chinese NSCLC patients as well as examine the therapeutic efficacy of crizotinib in patients with different *ROS1* fusion partners.

## 2 Materials and methods

### 2.1 Patient information and sample collection

A series of 17 158 consecutive clinical lung cancer cases were analyzed using comprehensive genomic profiling targeting 400+ cancer‐relevant genes containing all exons/flanking intronic regions and select introns (introns 31–35) as well as select exons and introns of *ROS1* fusion partner genes, including *CD74, SDC4, EZR*, and *SLC34A2*. Genomic profiling was performed by a Clinical Laboratory Improvement Amendments‐certified, College of American Pathologists‐accredited laboratory (Nanjing Geneseeq Technology, Jiangsu, China), as previously described [10]. Written informed consent was collected from each patient upon sample collection, according to the protocols approved by the ethics committee of The First Affiliated Hospital of Zhengzhou University, Henan, China. The study was conducted in compliance with the Declaration of Helsinki.

We identified patients with *ROS1* fusions in the laboratory information management system (LIMS) database using a natural language search program. For those cases, relevant demographic and clinical data were extracted from the database, including age, gender, date of diagnosis, histology type, pathological stage, and evaluation of treatment responses per the reports of the clinical investigators. For tumor tissue samples, the pathologic diagnosis and tumor content for each case were confirmed by pathologists. A total of 8–10 mL of peripheral blood was collected in EDTA‐coated tubes (BD, Franklin Lakes, NJ, USA). Within 2 h of collection, samples were centrifuged at 1800 ***g*** for 10 min to separate the plasma from white blood cells. Plasma samples were used for circulating tumor DNA extraction, while white blood cells were used for genomic DNA extraction (germline control).

### 2.2 DNA extraction and targeted enrichment

Circulating tumor DNA from plasma was purified using the Circulating Nucleic Acid Kit (Qiagen, Hilden, Germany) following the manufacturer's protocol. Genomic DNA from the white blood cells was extracted using the DNeasy Blood & Tissue Kit (Qiagen), while genomic DNA from formalin‐fixed paraffin‐embedded (FFPE) tissue was purified using the QIAamp DNA FFPE Tissue Kit (Qiagen). All DNA was quantified using the dsDNA HS Assay Kit on a Qubit Fluorometer (Life Technologies, Waltham, MA, USA). Sequencing libraries were prepared using the KAPA Hyper Prep Kit (KAPA Biosystems, Roche, Basel, Switzerland), as described previously [10, 11, 12]. Indexed DNA libraries were pooled for probe‐based hybridization capture of the targeted gene regions covering over 400 cancer‐related genes for all solid tumors, all of which contain all exons of *ROS1* and select introns for the detection of *ROS1* fusions.

### 2.3 Sequence data processing

Sequencing was performed using the Illumina HiSeq4000 platform, followed by data analysis as previously described [11, 13]. In brief, sequencing data were analyzed by Trimmomatic [14] to remove low‐quality (quality < 15) or N bases and were then mapped to the human reference genome, hg19, using the burrows–wheeler aligner (https://github.com/lh3/bwa/tree/master/bwakit). PCR duplicates were removed by picard (available at: https://broadinstitute.github.io/picard/). The Genome Analysis Toolkit (gatk) (https://software.broadinstitute.org/gatk/) was used to perform local realignments around indels and base quality reassurance. Gene fusions were identified by factera [15]. SNPs and indels were analyzed by varscan2 [16] and HaplotypeCaller/UnifiedGenotyper in gatk, with the mutant allele frequency (AF) cutoff at 0.5% for tissue samples, 0.1% for cell‐free DNA samples, and a minimum of three unique mutant reads. Common SNPs were excluded if they were present in > 1% population frequency in the 1000 Genomes Project or the Exome Aggregation Consortium (ExAC) 65 000 exomes database. The resulting mutation list was further filtered using an in‐house list of recurrent artifacts based on a normal pool of whole‐blood samples.

### 2.4 Statistical analysis

Categorical variables were compared between mutation carriers and non‐carriers using the Fisher's exact test. The Kaplan–Meier method was used for survival analyses, and statistical significance was assessed using the logrank test. A two‐tailed *P*‐value < 0.05 was considered statistically significant. All statistical analyses were performed using r version 3.4.5 (Boston, MA, USA).

## 3 Results

### 3.1 Patient characteristics

From December 2016 to November 2019, a total of 17 158 individual clinical NSCLCs were successfully evaluated by comprehensive genomic profiling using hybrid capture‐based NGS, as previously described [10]. Lung cancer tumor samples and liquid biopsies, if applicable, were compared to matched normal whole‐blood controls. Approximately 87% of NSCLCs examined were lung adenocarcinoma (LUAC, *n* = 14 927), 10% were lung squamous cell carcinoma (*n* = 1717), and the remainder (3%) were of either mixed or unknown histology. A total of 258 unique patients (1.5%, 258/17 158) were identified with a *ROS1* gene rearrangement, including both newly diagnosed and relapsed patients on prior therapies. The majority (90%) of the cohort included 3′ *ROS1*‐rearrangment events, while 5′ *ROS1* fusion events were present in about 10% of the cohort (*n* = 28), and mainly involved *CD74*, intergenic regions, or rare partner genes. It is of clinical interest to determine if such 5′ *ROS1* gene arrangements would eventually result in a functional fusion protein. However, it was unfortunate that we did not obtain such evidence.

Patients' demographic and clinical data are summarized in Table 1. The median age of the cohort was 54 years old (range: 26–96 years old). Approximately 60% (154/258) of patients were female, and the majority of the cohort contained adenocarcinoma (86%, 223/258). The majority (73%) of the cohort were pathologic stage III/IV with very few patients being stage I/II (4%). Such an observation was partially because early stage cancer patients are more eligible for curative treatment regimens as opposed to systemic therapy including targeted therapies. Approximately 25% of the cohort was missing the stage data. Approximately 12% of the *ROS1+* cohort underwent surgery as the first‐line treatment, while 40% received frontline chemotherapy. A total of 68 patients had crizotinib exposure, including 22 cases that were confirmed of frontline crizotinib therapy. Approximately 37% of the *ROS1+* patients had no treatment data in our database.

**Table 1:** A summary of *ROS1+* NSCLC patients' demographic and clinical characteristics.

Characteristics	*ROS1*‐rearranged NSCLC (*n*, %)
No. of patients	258
Gender	Female (154, 60%)
	Male (104, 40%)
Age (media, years)	54 (range: 26–96)
Histology	Adenocarcinoma (223, 86%)
	Squamous cell carcinoma (3, 2%)
	Large‐cell carcinoma (1)
	Mixed histology (1)
	Uncharacterized (30, 12%)
Pathologic stage	I/II (9, 4%)
	III/IV (189, 73%)
	Unknown (60, 23%)
Frontline treatment	Surgery (31, 12%)
	Chemotherapy (102, 40%)
	Crizotinib (22, 9%)
	Other TKIs (6, 2%)
	Unknown (97, 37%)
Crizotinib exposure	First‐line (22)
	Second‐line and beyond (46)

### 3.2 Identification of *ROS1* fusion partners

We identified a total of 258 *ROS1*‐rearranged NSCLC patients. Sixty‐six patients were tested for *ROS1* fusion using only liquid biopsies, including 40 cases of plasma, 25 cases of malignant pleural effusion, and one case with both plasma and pleural effusion samples tested. The AF in positive tumor specimens (FFPE or frozen tumor tissues) was significantly higher than that of liquid biopsies (median AF: 11.5% vs. 1.9%, *P* < 0.001) (Fig. S1). In particular, the AF in pleural effusion specimen was higher than that of plasma (median AF: 5.5% vs. 1.1%, *P* = 0.02; Fig. S1).

**Fig. 1:**
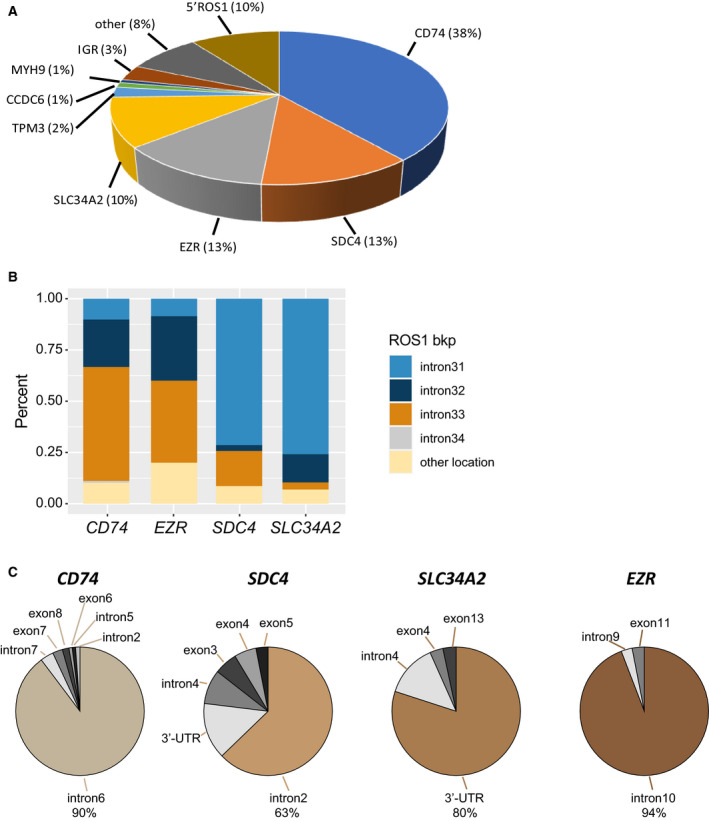
*ROS1* rearrangements in NSCLC. (A) Frequency of *ROS1* fusion variants. (B) Distribution of fusion breakpoint positions in the most common *ROS1* fusion pairs, including *CD74‐ROS1, SDC4‐ROS1, EZR‐ROS1*, and *SLC34A2‐ROS1*. (C) Distribution of breakpoint locations for *ROS1* fusion partner genes.

Fusion partners detected in this cohort included the well‐documented ones, including *CD74, SDC4, EZR*, and *SLC34A2*, as well as other less frequent ones, such as *TPM3, CCDC6*, and *MYH9* (Fig. 1A). *CD74* (38%) was the most common *ROS1* fusion partner, followed by *EZR* (13%), *SDC4* (13%), and *SLC34A2* (10%). *ROS1* rearrangement most frequently occurred in *ROS1* introns 31, 32, 33, and 34, while less frequently in other exons and introns, including introns 17, 26, 28, and 30. For *CD74‐ROS1* fusions, *ROS1* intron 33 was the predominant breakpoint location, while *ROS1* intron 31 was most abundant breakpoint location in *EZR* and *SLC34A2* fusions (Fig. 1B). *ROS1* most frequently fused to intron 6 of *CD74*, intron 2 of *SDC4*, and intron 10 of *EZR*, while the 3′ UTR of *SLC34A2* was disrupted in the majority of *SLC34A2‐ROS1* pairs (Fig. 1C).

Aside from the well‐known *ROS1* fusion partners, we also identified multiple genes involved in rearrangement with *ROS1*, including *TPM3*, *CCDC6*, and *MYH9* (Fig. 1A and Table S1). Such rearrangements were observed at a relatively lower frequency but were recurrent. Unique *ROS1* fusion events were also observed in the cohort at an incidence of 8% (*n* = 22, Table S1) and involved *LRIG3, WNK1*, and *SLC2A4RG*, among other genes that have previously not documented as *ROS1* fusion partners in the literature. Further research is required to validate such fusions in additional patients. In addition to the low frequency fusions, we also observed 10 fusion events that involved the intergenic regions of genes (Fig. 1A and Table S1), including *FAM65B* and *GRIK2*. It is clinically important to validate the presence of a functional *ROS1* fusion protein in these cases.

### 3.3 Resistance mechanisms to crizotinib in *ROS1*‐positive patients

**Fig. 2:**
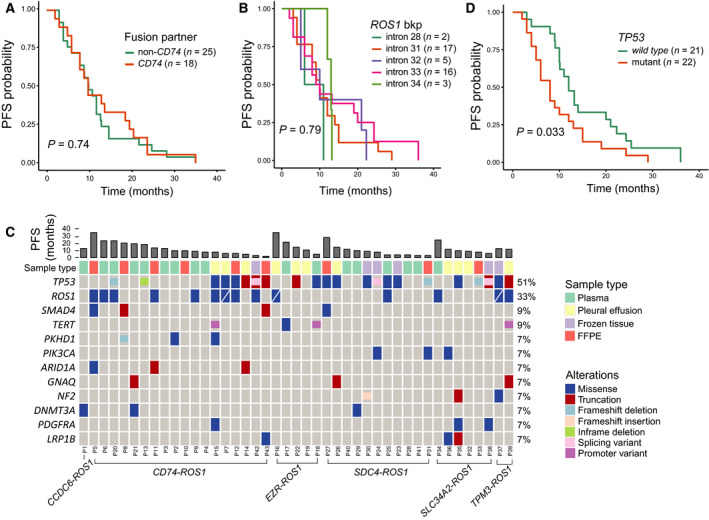
Crizotinib PFS of *ROS1+* NSCLC and somatic mutation profiles of post‐crizotinib specimen. (A) Crizotinib PFS data for *CD74‐ROS1* fusion pairs vs. non‐*CD74‐ROS1* fusion pairs. (B) Crizotinib PFS data for fusion variants with breakpoints in different *ROS1* introns. (C) Mutation profiles of post‐crizotinib samples from 43 *ROS1+* NSCLC patients. Genes with more than two occurrences of aberrations were shown in the plot. ‘/’ indicates *ROS1* mutations other than *ROS1* G2032R. (D) Crizotinib PFS data of *TP53‐*mutant *ROS1+* patients as compared to *TP53 wild‐type* patients.

Crizotinib has demonstrated favorable clinical efficacy in *ROS1*‐rearranged NSCLC patients. A total of 68 patients were once treated with crizotinib including 22 cases of first‐line use. Forty‐three patients had crizotinib progression‐free survival (PFS) information in our database, and their demographic and clinical data are provided in Table S2. The PFS data of the remaining 25 patients were unfortunately unavailable for further analysis. Our data showed that there was no significant difference in PFS between *CD74‐ROS1* patients and non‐*CD74‐ROS1* patients although the median PFS in the non‐*CD74‐ROS1* subgroup was slightly longer than that of *CD74‐ROS1* patients (median PFS: 10.6 vs. 10.0 months, Fig. 2A and Table S2). This finding could be attributed to both the small cohort size and the heterogeneity in the lines of therapy. Moreover, we also questioned whether survival differed among *ROS1* fusion variants with breakpoints in different *ROS1* introns. However, no significant differences were observed among those patient subgroups (Fig. 2B).

We further investigated the genetic profiles of post‐crizotinib samples in the PFS cohort. Roughly 65% (28/43) of post‐crizotinib samples were liquid biopsies including plasma and malignant pleural effusion specimen. The remaining 35% (15/43) of the cohort had FFPE or tumor tissue samples derived from local primary lung lesion. As shown in Fig. 2C, *TP53* was the most frequently mutated gene (51%) in *ROS1‐*rearranged patients with relapsed disease. Notably, *TP53* mutations were significantly associated with shorter crizotinib PFS (median PFS: *wild‐type* = 12 months, *n* = 21 vs. mutant = 8 months, *n* = 22, *P* = 0.033, Fig. 2D). Furthermore, we also identified 14 patients (33%) who harbored *ROS1* point mutations, of which *ROS1* G2032R, the most common mechanism of crizotinib resistance in *ROS1‐*positive NSCLC, was present in 11 patients (Fig. 2C). Less frequent *ROS1* mutations, including *ROS1* G1957A, S1986F, and G2086F, were detected in another three patients (Fig. 2C and Table S2), while mutated *PIK3CA* was also detected in an additional three patients (7%, Fig. 2C and Table S2). While it is interesting to know how different patients progressed, we lack sufficient information of the specific lesions' drug responses required for further analysis.

Moreover, it is clinically interesting to determine if primary drug resistance conferred rapid disease progression in the three patients who had PFS less than 3 months. A *ROS1* G2032R mutation was detected in the post‐crizotinib sample of one patient (P42), but it was difficult to determine disease progression due to a lack of pretreatment specimen. No known resistance mechanisms were detected in the other two patients. In addition, 11 patients received next‐generation *ROS1* TKIs, including lorlatinib, ceritinib, and cabozantinib after they developed resistance to crizotinib. According to their medical records, five patients derived a durable clinical benefit from TKI treatment, while the remaining six patients quickly progressed on the specified TKIs (Table S2).

## 4 Discussion

The discovery of driver oncogenes with aberrant tyrosine kinase activation, such as *ALK, ROS1, RET*, or *NTRK1* gene rearrangement in cancers, continues to change the therapeutic strategies and treatment regimens accompanied with the development of targeted therapies. *ROS1* gene rearrangement, originally described in glioblastomas (*FIG‐ROS1*) [17], is reported at 2% in NSCLC [2] and up to 3.3% in LUAC [18]. Here, we report an overall prevalence of *ROS1* fusions of approximately 1.5% in a large cohort of Chinese NSCLC patients. LUAC represents the most predominant histological subtype of NSCLC in China where the proportion ranges from 43% to 46% in different geographic areas, including Beijing [19, 20]. The higher percentage of adenocarcinoma (87%) observed in our database can be largely explained by a bias toward relevant targeted therapies and clinical trials relating to patient selection for NGS testing. Diverse *ROS1* fusion partners were identified, including common genes such as *CD74, EZR, SDC4,* and *SLC34A2*, less common genes such as *TPM3* and *CCDC6*, and rare unique cases (8%) involving genes such as *WNK1* and *SLC2A4RG*, among others. *CD74* was the most common *ROS1* fusion partner at a frequency similar to that reported previously [1, 21]. Rare fusion pairs remain clinically interesting, but further research is needed to confirm these observations in preclinical studies and clinical cases.

Targeted inhibition of the aberrant ROS1 kinase with crizotinib is associated with increased PFS and improved quality‐of‐life measures [3, 5]. However, patients with concomitant *TP53* mutations had poorer survival than the *TP53 wild‐type* subset. Li *et al*. [22] reported that non‐*CD74‐ROS1* fusions tended to have longer PFS than the *CD74‐ROS1* group when treated with crizotinib. In our study, the PFS of non‐*CD74‐ROS1* pairs and *CD74‐ROS1* pairs was not significantly different, possibly due to the limited cohort size and high degree of heterogeneity in patients' baseline characteristics. Nor was there a significant difference among *ROS1* fusion variants with breakpoints in different *ROS1* introns.

In addition to crizotinib, other TKIs have shown clinically meaningful and durable responses in *ROS1*‐rearranged NSCLC patients, including ceritinib, lorlatinib, and entrectinib, which have been shown to have better intracranial effects compared to crizotinib [23, 24]. In particular, entrectinib, an orally administered selective inhibitor of ROS1/NTRK/ALK, was demonstrated to be more potent compared to crizotinib and was designed to penetrate the blood–brain barrier, which is of vital clinical importance as the central nervous system (CNS) is the first and sole site of progression in almost half of patients with ROS1 fusion‐positive NSCLC who are treated with crizotinib [25, 26]. Drilon *et al*. [23] reported an ORR of 77% in a cohort of ROS1‐rearranged NSCLC patients undergoing frontline treatment of entrectinib, demonstrating both favorable systemic and intracranial activities. Collectively, these findings have broadened the therapeutic options for *ROS1‐*positive patients, regardless of CNS metastases at baseline.

Similar to the findings of a previous study [25], this study found that the *ROS1* G2032R mutation was the most frequently identified resistance mechanism to crizotinib. In particular, one *SLC34A2‐ROS1* patient who exhibited the *ROS1* G2032R mutation following crizotinib treatment achieved a durable partial response to cabozantinib, which rendered the possibility of taking cabozantinib to overcome the crizotinib resistance in the other eight G2032R‐positive patients. However, the potential for failure also exists, as reported by Guisier *et al*. [27]. Notably, cabozantinib has been shown to be associated with higher toxicity compared with crizotinib and is therefore limited as a therapeutic agent for some patients [28, 29]. Taletrectinib, a next‐generation ROS/TRK inhibitor, was reported to potently inhibit *ROS1* G2032R cells *in vitro*. A recent US phase I study also observed preliminary activity of taletrectinib in crizotinib‐refractory *ROS1+* NSCLC patients [30]. Moreover, we identified one *EZR‐ROS1* fusion‐positive case who exhibited the *ROS1* S1986F mutation in a crizotinib‐resistant sample. Previous studies showed that the *EZR‐ROS1* S1986F variant was sensitive to lorlatinib but not ceritinib *in vitro* [28], and therefore, the patient could benefit from lorlatinib following progression on crizotinib. In addition to *ROS1* G2032R and S1986F mutations, the *ROS1* G1957A mutation was also detected in one *CD74‐ROS1* patient previously treated with crizotinib. Such a finding has not previously been documented in the literature, and further research is needed to validate the oncogenicity of this substitution, or whether it confers crizotinib resistance.

It is worth noting that the bypass mechanism through PI3K signaling was observed in three patients with different fusion partners. Our findings reinforced a previous report by Xu *et al*. [31] that the activation of the PI3K pathway leads to acquired crizotinib resistance. It is also important to note that the mechanisms of crizotinib resistance remained unclear in approximately 60% of the post‐crizotinib cohort.

## 5 Conclusions

Using NGS testing, this study found that the prevalence of *ROS1* fusions in a large NSCLC cohort was 1.5%, including most frequent fusion partners and rare *ROS1* fusion pairs. Crizotinib has demonstrated robust response in treating patients with *ROS1*‐rearranged NSCLC. Recent advances in targeting the ROS1 tyrosine kinase using TKIs such as lorlatinib, cabozantinib, entrectinib, and taletrectinib have expanded the treatment options for the *ROS1+* population.

## Conflict of interest

Qiuxiang Ou, Xiaoling Tong, and Xue Wu are the employees of Geneseeq Technology Inc., Canada. The remaining authors have nothing to declare.

## Author contributions

MC and YH conceived and designed the study. PL and JZ collected the data. QO and XT analyzed the data. RZ, ND, and XW provided the resources for the study. WL and GJ supervised the study. MC, YH, QO, and GJ wrote the manuscript. All authors read and approved the final manuscript.

## Ethics approval

This study was approved by the Institutional Review Board/Ethics Committee of the First Affiliated Hospital of Zhengzhou University, Henan, China.

## Consent to participate

Written informed consent was obtained from each patient upon sample collection and for publication of the study.

## Supporting information


**Fig. S1.** The comparison of allele frequency of *ROS1* gene fusion in different sample categories subject to next‐generation sequencing.

**Table S1.** Fusion details of rare *ROS1* fusion events.

**Table S2.** The demographic and clinical data of a subset of the 43 *ROS1 + *patients who progressed on crizotinib.
