# Supplementary figures and images for: Molecular and clinicopathological characteristics of ROS1‐rearranged non‐small‐cell lung cancers identified by next‐generation sequencing
Source: Mol Oncol. 2020 Sep 14;14(11):2787–95. doi: 10.1002/1878-0261.12789 (PMC7607175; doi:10.1002/1878-0261.12789)

Supplementary Figure S1

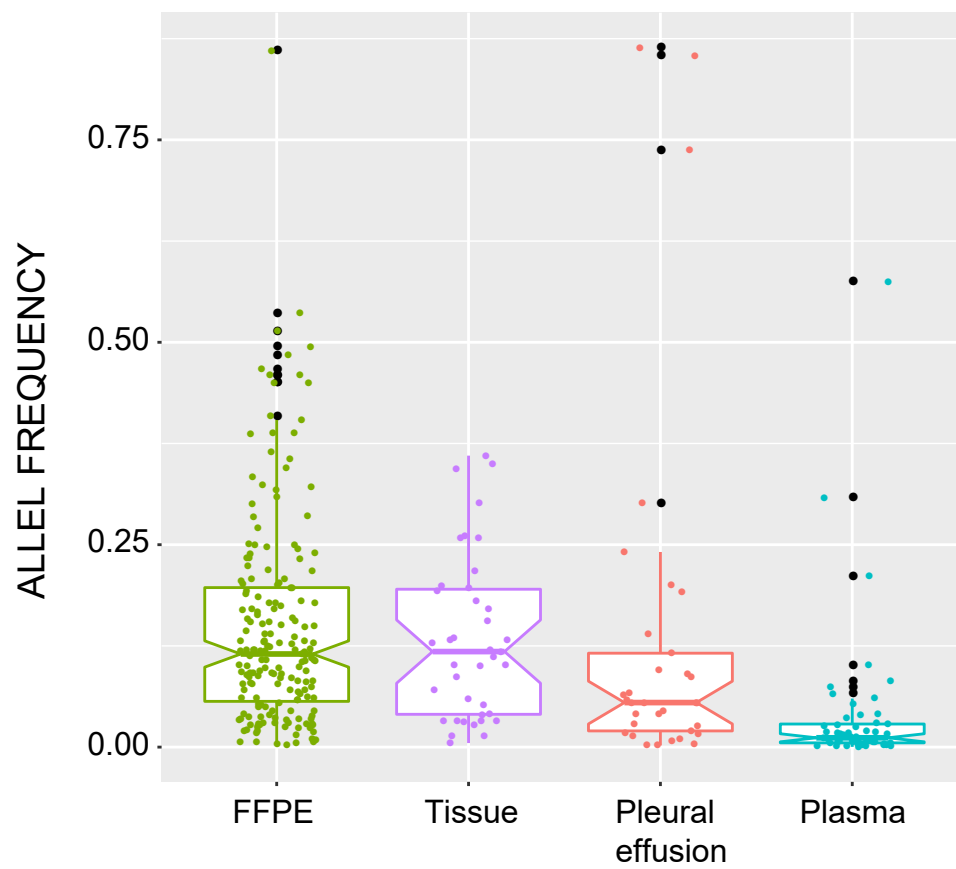

Supplement: Supplementary file 1 — Fig. S1. The comparison of allele frequency of ROS1 gene fusion in different sample categories subject to next‐generation sequencing. [file MOL2-14-2787-s001.pdf]
